# Changes in Self-Reported Web-Based Gambling Activity During the COVID-19 Pandemic: Cross-sectional Study

**DOI:** 10.2196/30747

**Published:** 2021-11-03

**Authors:** Emma Claesdotter-Knutsson, Anders Håkansson

**Affiliations:** 1 Child and Adolescent Psychiatry Department of Clinical Sciences Lund University Lund Sweden; 2 Psychiatry, Department of Clinical Sciences Faculty of Medicine Lund University Lund Sweden

**Keywords:** COVID-19, pandemic, web-based gambling, psychological distress, gender

## Abstract

**Background:**

The COVID‑19 pandemic has affected not only somatic health with over 3.7 million deaths worldwide, but also has had a huge impact on psychological health, creating what amounts to a mental health crisis. The negative effect of the pandemic on traditional addictions is well described and concerning, and the same has been seen for gambling.

**Objective:**

This study explores self-reported web-based gambling behavior during the COVID‑19 pandemic in Sweden. We investigated overall changes, but also changes in specific web-based gambling types, and whether they are associated with certain risk factors or lifestyle changes.

**Methods:**

Our study is based on an anonymous web-based survey of web panel participants in Sweden (N=1501) designed to study a range of behavioral changes during the COVID‑19 pandemic. Increases in gambling were analyzed using logistic regression models against sociodemographic data and psychological distress.

**Results:**

The majority of the respondents who gambled reported no changes in their gambling habits during the COVID‑19 pandemic. We found significant associations with the problem gambling severity index (PGSI), the Kessler score (indicating psychological distress), employment status, changes in alcohol habits, and self-exclusion when looking at overall changes in gambling activity in the pandemic. In the subgroup that reported an increase in gambling activity, we found an association with both the PGSI and Kessler scores. The PGSI score was also an independent predictor for all specific web-based gambling (horses, sports, poker, and casino) whereas the Kessler score only had a significant impact on changes in casino gambling. In addition, male gender was an independent predictor for gambling on sports and casino gambling.

**Conclusions:**

The majority of respondents who gambled reported no changes in their gambling activity during the COVID‑19 pandemic. The group that reported an increase in overall gambling activity during the COVID-19 pandemic represent a group with gambling problems and psychological distress. The group that reported increased sports and casino gambling were often male, and this group seemed to experience more psychological distress.

## Introduction

The first case of COVID‑19 was detected in December 2019 in Wuhan, China [1] and then the virus spread rapidly, reaching Europe in early 2020 and Sweden in January 2020 [2]. The COVID‑19 pandemic has had an enormous effect on mental health [3-5]. Research has confirmed increasing rates of stress, depression, addiction, anxiety, and other psychiatric disorders during the pandemic [6,7].

Virus protection strategies in Sweden have differed from those in other countries and not included lockdown or stay-at-home orders, but rather recommendations. Most work places encouraged their workers to work from home and regulations in opening hours have been in effect for restaurants and shopping malls, leading people in general to spend a lot more time indoors with possible access to screens, and several studies have reported increased screen time during the pandemic [8-11]. Excessive screen time use has been shown to be associated with a range of negative mental health outcomes such as anxiety and depression [12-14].

Historically in economic crises, when people experienced stress due to, for example, isolation, gambling activity per se increased, and so did gambling problems [15-17], but recent studies on potential changes in gambling activity during the COVID‑19 pandemic have reported different changes in behavior [18]. One possible explanation might be the restrictions in place in the field of study, along with differences in study populations. Auer et al [19] and Lindner et al [20] found a substantial decrease in overall gambling activity, especially in gambling, where there were far fewer betting opportunities because of cancelled or postponed sports events such as football leagues.

This was also reported in an Italian study conducted during the lockdown, when gamblers reported not only a decrease in gambling but also the relief they felt because the restrictions meant they could not gamble as before [21]. Auer et al [19] confirmed this situation in a population of Swedish, German, Finnish, and Norwegian citizens, who reported no increase in gambling activity. Our research group, focusing on COVID-19–related changes in gambling in a Swedish setting, observed increased gambling activity in a subgroup of high-risk gamblers [22,23]. Lindner et al [20] observed the same pattern among high-risk gamblers and Frisone et al [24] observed this among moderate-risk gamblers.

Early during the pandemic, the Swedish government voiced its concerns about an anticipated increase in gambling owing to rising anxiety, boredom, and depression prevalence expected as the pandemic accelerated [20]. Social distancing to prevent virus transmission was thought likely to encourage gambling [20]. To prevent this, the Swedish government introduced a temporary legislation (Förordning 2020:495) to govern the Swedish gambling market, including a deposit limit at web-based casinos, of Swedish kronor (SEK) 5000 (US $580.48) a week (per gambling provider) and obligatory time limits because of concerns in the early stages of the outbreak that social distancing would increase web-based activities such as gambling and, by extension, the problems associated with gambling. Other countries followed suit [20,23].

Gambling is strongly associated with comorbid disorders, including alcohol use disorder, depression, substance use disorders, nicotine dependence, anxiety disorders, and antisocial personality disorder [25,26]. Gambling is a potentially addictive behavior; gambling disorder is an addiction diagnosis given the same status as alcohol and drug addiction [27,28]. Web-based gambling, known for its autonomy, accessibility, and speed of play, is considered a higher-risk form of gambling, which easily leads to addiction [29,30].

In our study, we explored self-reported web-based gambling behavior during the COVID‑19 pandemic in Sweden. We considered overall changes, along with changes for specific types of gambling, and whether potential changes in gambling may be associated with specific risk factors such as level of education, employment status, disposable income, and psychological distress.

## Methods

### Setting

The first wave of the COVID‑19 pandemic in Sweden was in spring 2020. After a decline in virus transmission in the summer, there was a second wave in the autumn of 2020. In the first few months of 2021, there was a third wave of virus transmission and a further increase in the disease burden of hospitalizations (Swedish Public Health Agency 2021). This cross-sectional study is based on a self-report web-based survey carried out in Sweden in March 2021, during the third wave of the COVID‑19 pandemic in the country. At the time of this study, upper-secondary schools in Sweden had resumed in-person teaching, but the nationwide COVID-19 restrictions on leisure activities and gatherings of more than 8 people were still in effect.

### Participants

We consulted the market survey company Ipsos and used its internet-based web survey panel. We have previously used the Ipsos web panel for internet-based surveys for research purposes [30]. Respondents from the general population, aged 16 years and above, from the Ipsos web panel were invited with the information that the survey would address “computer gaming, gambling for money, and other behavioural patterns in Sweden during Covid-19—Association of mental health, social situation, and attitudes to the pandemic.” The language used in the survey was Swedish. Respondents could only access the survey once they had provided electronic informed consent. Most Ipsos surveys are worth 1 point to respondents, and each point is worth about €1 (US $1.16) when redeemed as goods or services. In this study, invitations were sent until 1500 complete answers were obtained. In addition, in this study, the final distribution of age groups, gender, and geographical location (region) was compared by Ipsos to the general population, such that the data set was weighted in accordance with a summarized weighting score derived from these 3 variables. The survey was halted when the final sample consisted of 1501 individuals. The study was carried out between March 19 and 29, 2021. The study was reviewed and approved by the Swedish Ethical Review Board (File: 2021/00369).

### Measures

The basic sociodemographic variables included gender (female or male), age (divided into 2 age groups of 16-24 years and ≥25 years), monthly income (divided into 3 groups of SEK 10,000-20,000 [US $1164.40-$2328.81], SEK 20,000-40,000 [US $2328.81-$4657.62], or >SEK 40,000 [>US $4657.62]), level of education (university, upper-secondary school [ages 16-19 years], compulsory school [ages 6-16 years], or other), and employment status (studying, employed, unemployed, retired, or other). The questionnaire continued with questions about changes in personal behavior during the COVID‑19 pandemic (“since these changes in Sweden started”): the time they spent at home (much more, slightly more, unchanged, or less time at home) and how much alcohol they consumed (more, less, unchanged, or “I don’t drink, now nor before”). The questionnaire continued with a general question about their web-based gambling on horse racing, sports, poker, and casino games (more, less, unchanged, or “I don’t gamble, now or before”). This was followed by questions for each type of web-based gambling in turn (horses, sports, poker, and casinos). A question was asked about self-exclusion from gambling (gambling breaks) using spelpaus.se, a national self-exclusion scheme that covers all forms of licensed gambling, which has been available since January 1, 2019 (yes, no, or prefer not to answer) [30].

Psychological distress was measured using the Kessler-6 scale to describe symptoms of depressed mood and anxiety [31]. It is a 6-item scale of symptoms perceived in the preceding 6 months, with 6 questions about depressive and anxiety-related symptoms with options ranging from “not at all” to “all the time,” including a “cannot answer/prefer not to say” response. For the Kessler-6 scale, the scores of 0-4 for each question were summed, and a total score of ≥5 was classified as at least moderate psychological distress.

The level of potential gambling problems was measured with the 9-item problem gambling severity index (PGSI) [32], where each statement addresses the preceding 12 months, with options including “never,” “sometimes,” “most of the time,” and “almost always.” For the PGSI scale, the scores of 0-3 for each question were summed: a total score of 0 indicated no problem with gambling; 1-2, a certain risk of gambling problems; 3-7, a moderate risk of gambling problems; and ≥8 indicated gambling problems.

### Statistical Analysis

The reporting of prevalence measures and group-wise comparisons related to the weighted data and statistical tests were applied using the chi-square test, whereas binary logistic regression analyses were carried out using the unweighted data. Binary logistic regressions were carried out with each increased change in gambling (yes/no) as the dependent variable to study potential–independent variables associated with the outcome. In a subsequent step, greater changes in specific forms of gambling (horses, sports, poker, and casino) were established as outcomes. For all models, odds ratios with 95% CIs are presented. SPSS (version 25.0) was used for all statistical analyses.

### Availability of Data and Materials

The data sets used and analyzed in the current study are available from the corresponding author on reasonable request.

### Ethics Approval and Consent to Participate

All procedures performed in this study involving human participants were in accordance with the ethical standards of the national research committee and with the tenets of the 1964 Helsinki declaration and its later amendments or comparable ethical standards.

### Patient Consent for Publication

Informed consent was obtained from all individual participants included in the study.

### Informed Consent

The manuscript does not contain any individual person’s data in any form.

## Results

### Descriptive Data on Changes in Web-Based Gambling Activity and Type of Gambling

The descriptive data of the sample are shown in Table 1 (weighted data). Of the 1501 respondents, 29% (n=437) responded they had never gambled and were therefore excluded from further analysis. Of the remaining 1064 who were further assessed, 56% (n=596) were male and 44% (n=468) were female. The distribution regarding the different types of web-based gambling activity is described in Table 1.

**Table 1:** Study population by total and self-reported changes in web-based gambling activity (weighted) (N=1501).

		Changes in gambling activity				
		Total, n (%)	Horses, n (%)	Casino, n (%)	Poker, n (%)	Sports, n (%)
Never gambled		437 (29.1)	1073 (71.5)	1122 (74.8)	1264 (84.2)	1007 (67.1)
Study population		1064 (70.9)	428 (28.5)	379 (25.2)	237 (18.8)	494 (32.9)
**Gender**						
	Female	468 (44.0)	150 (35.1)	123 (32.3)	56 (23.5)	140 (28.4)
	Male	596 (56.0)	278 (64.9)	256 (67.7)	181 (76.5)	354 (71.6)

Based on the findings, approximately 10% (n=111) of the participants reported an increase in overall gambling, 82% (n=876) reported unchanged gambling activity, and 7% (n=77) reported a decrease in gambling (Table 2). The figures for increased activity by gambling type were similar at approximately 12% (11.0%-12.9%). The same was seen for the self-reported decrease in activity by type of gambling on horses, sports, and casinos at approximately 13% (13.2%-14.2%), but self-reported decrease in poker was 21% (Table 2).

**Table 2:** Changes in web-based gambling activity overall and by gambling type (weighted).

Changes	Change in gambling, n (%)				
	Overall (n=1064)	On horses (n=428)	On casino (n=380)	On poker (n=237)	On sports (n=494)

Increased	111 (10.4)	47 (11.0)	49 (12.9)	28 (11.8)	56 (11.3)
Unchanged	876 (82.3)	322 (75.2)	277 (72.9)	160 (67.5)	373 (75.5)
Decreased	77 (7.2)	59 (13.8)	54 (14.2)	49 (20.7)	65 (13.2)

### Changes in Web-Based Gambling Activity by Demographic and Socioeconomic Factors

Table 3 presents the weighted data for changes in gambling by demographic and socioeconomic factors. We found significant differences in employment status (*P*<.001), PGSI (*P*<.001), Kessler score (*P*<.001), gambling paus (*P*<.001), as well as changes in alcohol habits (*P*<.001) (Table 3).

**Table 3:** Changes in gambling activity by demographic and socioeconomic characteristics (weighted).

		Increased (n=111), n (%)	Unchanged (n=876), n (%)	Decreased (n=77), n (%)	*P* value		Total (N=1064), n (%)	
**Gender**						.10		
	Female	39 (34.9)	398 (45.4)	32 (41.4)			468 (44.0)	
	Male	72 (65.1)	478 (54.6)	45 (58.6)			596 (56.0)	
**Age groups (years)**						.07		
	16-24	19 (17.3)	96 (11.0)	13 (16.4)			128 (12.0)	
	≥25	92 (82.7)	780 (89.0)	64 (83.6)			936 (88.0)	
**Level of education**						.51		
	Primary school	55 (49.2)	402 (46.0)	41 (52.9)			498 (46.8)	
	Secondary school	45 (40.4)	364 (41.5)	28 (36.5)			437 (41.0)	
	University	9 (7.7)	82 (9.3)	3 (4.5)			94 (8.8)	
	Other	3 (2.7)	28 (3.2)	5 (6.1)			36 (3.3)	
**Employment status**						<.001^a^		
	Studying	21 (18.9)	86 (9.9)	12 (16.1)			120 (11.3)	
	Employed	63 (56.3)	499 (57.0)	50 (65.0)			612 (57.5)	
	Unemployed	16 (14.2)	44 (5.1)	4 (4.6)			63 (6.0)	
	Retired	10 (8.9)	225 (25.7)	11 (14.3)			246 (23.1)	
	Other	2 (1.9)	21 (2.4)	0 (0)			23 (2.2)	
**Disposable income**						.25		
	≥SEK 40,000 (US $4657.62)	35 (31.8)	241 (27.5)	29 (38.1)			305 (28.7)	
	SEK 20,000-40,000 (US $2328.81-$4657.62)	60 (54.1)	474 (54.2)	37 (48.2)			572 (53.7)	
	≤SEK 10,000-20,000 (US $1164.40-2328.81)	16 (14.2)	161 (18.3)	11 (13.7)			187 (17.6)	
**Problem Gambling Severity Index**						<.001^a^		
	No problem with gambling	25 (24.5)	600 (80.0)	41 (61.2)			666 (62.6)	
	Certain risk of gambling problems	17 (17.2)	86 (11.4)	14 (21.6)			117 (11.0)	
	Increased risk of gambling problems	34 (33.9)	55 (7.3)	7 (10.7)			96 (9.1)	
	Gambling problems	25 (24.4)	10 (1.4)	4 (6.5)			39 (3.7)	
**Kessler score**						<.001^a^		
	Score 0-4; no psychological distress	19 (16.7)	443 (51.1)	25 (34.7)			487 (45.8)	
	Score 5-24; psychological distress	92 (83.2)	423 (48.9)	48 (65.3)			563 (52.9)	
**Gambling pause**						<.001^a^		
	Yes	21 (18.2)	24 (2.8)	12 (14.9)			51 (5.2)	
	No	91 (81.7)	846 (9.7)	65 (85.1)			1000 (94.8)	
**Time at home**						.11		
	Much more	76 (67.8)	496 (5.7)	44 (57.3)			614 (57.8)	
	Slightly more	29 (25.8)	258 (29.5)	20 (25.8)			307 (28.8)	
	Unchanged	7 (5.6)	119 (13.6)	12 (15.4)			137 (12.9)	
	Less time	1 (0.9)	4 (0.4)	1 (1.6)			6 (0.5)	
**Change in alcohol habits**						.001^a^		
	Yes more	26 (23.2)	74 (8.5)	8 (9.6)			105 (10.1)	
	Unchanged	38 (34.0)	530 (60.5)	24 (31.2)			592 (55.6)	
	Less alcohol	30 (27.3)	186 (21.2)	33 (43.2)			249 (23.4)	
	Does not drink	17 (15.5)	86 (9.9)	12 (16.0)			116 (10.9)	

^a^Statistically significant.

### Comparison of Increased Gambling in Different Outcomes

Multivariable analysis using binary logistic regression models of the potential predictors of increased gambling (yes vs no) is presented in Table 4 (unweighted data). Increased gambling was associated with all 3 categories of PGSI score, whether “Certain risk of gambling problems” (OR 4.40, 95% CI 2.17-8.93), “Increased risk of gambling problems” (OR 12.53, CI 6.54-24.04), or “Gambling problems” (OR 32.42, 95% CI 13.77-76.35). The PGSI for “Gambling problems” was also correlated with increased gambling in all specific forms of gambling: horses (OR 12.64, 95% CI 3.94-40.68), sports (OR 38.81, 95% CI 11.65-129.28), poker (OR 10.53, 95% CI 2.24-49.43), and casinos (OR 26.17, 95% CI 7.09-96.62). An increased change in gambling overall was associated with the >5 Kessler score (OR 2.62, 95% CI 1.39-4.91) and with increased casino gambling (OR 4.47, 95% CI 1.15-17.3). In addition, male gender is correlated with increased sports betting (OR 2.58, 95% CI 1.09-6.09) and casino gambling (OR 2.73, 95% CI 1.06-7.07). The correlation between “Does not drink” and increased sports betting was also significant (OR 3.95, 95% CI 1.02-15.33).

**Table 4:** Multivariable logistic regressions model with increased gambling as the outcome of interest (yes vs no).

			Increased gambling, odds ratio (95% CI)		Increased gambling on horses, odds ratio (95% CI)		Increased gambling on casino, odds ratio (95% CI)		Increased gambling on poker, odds ratio, (95% CI)		Increased gambling on sports, odds ratio (95% CI)
**Gender**											
	Female	1.00 (reference)		1.00 (reference)		1.00 (reference)		1.00 (reference)		1.00 (reference)	
	Male	1.46 (0.86-2.48)		0.84 (0.39-1.81)		2.58^a^ (1.09-6.09)		1.51 (0.50-4.59)		2.73^a^ (1.06-7.07)	
**Age groups (years)**											
	16-24	1.00 (reference)		1.00 (reference)		1.00 (reference)		1.00 (reference)		1.00 (reference)	
	≥25	1.09 (0.48-2.50)		2.09 (0.35-12.52)		0.43 (0.14-1.34)		0.91 (0.18-4.54)		0.67 (0.24-1.88)	
**Level of education**											
	Primary school	1.00 (reference)		1.00 (reference)		1.00 (reference)		—^b^		1.00 (reference)	
	Secondary school	0.73 (0.27-1.99)		0.80 (0.19-3.47)		1.62 (0.26-9.93)		—		2.39 (0.46-12.54)	
	University	1.18 (0.44-3.19)		0.85 (0.20-3.69)		1.69 (0.27-10.43)		—		2.82 (0.52-15.23)	
	Other	1.31 (0.27-6.36)		0.72 (0.05-9.64)		2.62 (0.15-45.97)		—		5.83 (0.38-89.42)	
**Employment status**											
	Studying	1.00 (reference)		1.00 (reference)		1.00 (reference)		1.00 (reference)		1.00 (reference)	
	Employed	0.61 (0.22-1.69)		0.38 (0.07-2.03)		0.41 (0.09-1.96)		0.49 (0.05-5.00)		0.50 (0.13-1.92)	
	Unemployed	1.79 (0.62-5.19)		1.59 (0.28-9.11)		2.84 (0.63-12.85)		0.89 (0.10-8.18)		0.86 (0.20-3.62)	
	Retired	0.64 (0.21-1.98)		0.73 (0.12-4.53)		0.51 (0.04-5.82)		—		0.79 (0.17-3.81)	
	Other	2.11 (0.37-12.06)		7.64 (0.70-83.40)		—		—		—	
**Disposable income**											
	≥SEK 40,000 (US $4657.62)	1.00 (reference)		1.00 (reference)		1.00 (reference)		1.00 (reference)		1.00 (reference)	
	SEK 20,000-40,000 (US $2328.81-$4657.62)	0.90 (0.42-1.95)		0.51 (0.19-1.36)		0.59 (0.15-2.33)		1.23 (0.27-5.58)		0.90 (0.28-2.89)	
	≤SEK 10,000-20,000 (US $1164.40-2328.81)	0.73 (0.26-2.04)		0.30 (0.08-1.21)		0.41 (0.06-2.65)		0.44 (0.04-5.01)		0.84 (0.17-4.09)	
**Problem Gambling Severity Index**											
	No problem with gambling	1.00 (reference)		1.00 (reference)		1.00 (reference)		1.00 (reference)		1.00 (reference)	
	Certain risk of gambling problems	4.40 (2.17-8.93)		4.01^a^ (1.26-12.81)		4.54^a^ (1.13-18.30)		7.49^a^ (1.52-36.78)		6.14^a^ (2.00-18.92)	
	Increased risk of gambling problems	12.5^a^ (6.54-24.04)		10.93^a^ (3.88-30.82)		6.02^a^ (1.68-21.56)		3.84 (0.80-18.51)		10.91^a^ (3.71-32.07)	
	Gambling problems	32.4^a^ (13.77-76.35)		12.64^a^ (3.93-40.68)		26.17^a^ (7.09-96.62)		10.53^a^ (2.24-49.43)		38.81^a^ (11.65-129.28)	
**Kessler score**											
	Score 0-4; no psychological distress	1.00 (reference)		1.00 (reference)		1.00 (reference)		1.00 (reference)		1.00 (reference)	
	Score 5-24; psychological distress	2.62^a^ (1.39-4.91)		2.06 (0.84-5.07)		4.47^a^ (1.15-17.30)		2.80 (0.69-11.74)		1.09 (0.46-2.57)	
**Gambling pause**											
	Yes	1.00 (reference)		1.00 (reference)		1.00 (reference)		1.00 (reference)		1.00 (reference)	
	No	1.14 (0.43-3.02)		0.77 (0.22-2.72)		1.66 (0.47-5.94)		0.92 (0.18-4.82)		2.48 (0.69-8.94)	
**Time at home**											
	Much more	1.00 (reference)		1.00 (reference)		1.00 (reference)		1.00 (reference)		1.00 (reference)	
	Slightly more	0.56 (0.30-1.04)		0.86 (0.36-2.05)		0.81 (0.29-2.20)		0.27 (0.07-1.12)		0.42 (0.16-1.07)	
	Unchanged	0.25 (0.08-0.77)		0.13 (0.01-1.18)		0.28 (0.05-1.76)		—		0.34 (0.08-1.49)	
	Less time	0.78 (0.04-13.93)		—		—		—		—	
**Change in alcohol habits**											
	Yes more	1.00 (reference)		1.00 (reference)		1.00 (reference)		1.00 (reference)		1.00 (reference)	
	Unchanged	0.66 (0.30-1.44)		0.28 (0.09-0.86)		0.72 (0.18-2.81)		0.18^a^ (0.03-0.98)		0.76 (0.24-2.40)	
	Less alcohol	0.77 (0.34-1.76)		0.53 (0.17-1.67)		1.45 (0.36-5.72)		0.24 (0.04-1.48)		0.62 (0.18-2.09)	
	Does not drink	1.31 (0.51-3.34)		1.22 (0.33-4.37)		1.32 (0.25-6.95)		2.07 (0.34-12.69)		3.95^a^ (1.02-15.33)	

^a^Significant correlations observed.

^b^—: not determined.

## Discussion

### Principal Findings

This cross-sectional study considers self-reported changes in web-based gambling in a general population aged 16 years and over during the third wave of the COVID‑19 pandemic in Sweden. We set out to study possible changes in gambling behavior and relate them to risk factors such as gender, level of education, employment status, disposable income, alcohol consumption, and psychological distress.

The majority of the respondents who gambled reported no changes in their gambling activity during the COVID‑19 pandemic. Regarding overall changes in gambling, we found significant associations with the PGSI (indicating the severity of the problem), the Kessler score (indicating psychological distress), employment status, changes in alcohol habits, and self-exclusion (gambling breaks). In the subgroup that reported an increased gambling activity, we found an association with both the PGSI score and the Kessler score. The PGSI score was also an independent predictor for all web-based gambling activities (horses, sports, poker, and casinos) whereas the Kessler score only had a statistically significant impact on changes in casino gambling. In addition, male gender was an independent predictor for sports betting and casino gambling.

### Changes in Web-Based Gambling Activity During the Pandemic

In a previous study conducted during the first wave of the COVID‑19 pandemic in 2020, we found a 4% self-reported increase in gambling [23], whereas in another study 7 months later, we found a 6% self-reported increase in gambling [22]. Surprisingly, Lindner et al [20], who also studied changes in gambling activity in Sweden during the first wave, found an overall decrease in gambling [20]. In this study conducted in March 2021 the majority (82%) of the respondents who reported that they gambled had not changed their gambling type during the pandemic, but we found a self-reported increase in gambling activity of 10%. Even though the studies are not fully compared because they use different subjects and this study is limited to web-based gambling only, one can nevertheless discern a consequent rise in gambling during the pandemic. This might be explained by people being more likely to change their behavior in the initial stages of the pandemic, similar to other times of crisis, but with time people tended to return to a stable, “normal” level, whether in gambling or other activities.

In the early stages of the pandemic, all sports betting events such as the top football leagues were cancelled [19]. Gradually, sporting events restarted, making sports betting easier. Another explanation for the increase in betting during the pandemic could be changes in society, such as restrictions on social events and restaurant opening hours, which implies that people were more likely to stay at home, where their screens—and gambling opportunities—were more readily available, thus affecting web-based gambling. The seemingly steady rise in self-reported gambling is something to follow as the pandemic continues.

### Characteristics Among Those Reporting an Increase in Specific Types of Gambling

The PGSI, Kessler score (psychological distress), employment status, gambling breaks, and changes in alcohol habits were all individually associated with changes in gambling—something that was not true for gender, age group, level of education, disposable income, and changes in time spent at home. The only factors that remained significantly associated with an overall increase in gambling were self-reported gambling problems and psychological distress.

Our findings are in line with those of previous findings that the minority who reported increased gambling during the COVID‑19 pandemic also reported increased gambling problems and greater psychological distress [18,20,23,33]. Problem gambling is known to be associated with mental health problems, adding to the impression that individuals who gambled more during the COVID‑19 pandemic were a vulnerable subgroup of the population. Increased casino gambling was the only form of gambling associated with psychological distress. Casino gambling is considered potentially highly addictive and is associated with problem gambling and personal debt [30,34,35]. Notably, at the onset of the COVID‑19 pandemic, both Lindner et al [20] and Håkansson [23] found that respondents who gambled using web-based casinos, rather than gambling less (as was the case for other types of gambling), increased their gambling activity.

Among respondents reporting an increase in sports betting and casino gambling, we found the traditionally male gender correlation [36,37]. This had not been true of sports betting in our previous study in April 2020, early in the COVID‑19 pandemic [23]. At that stage the sports betting market had shrunk—a plausible explanation for not seeing any gender differences in self-reported increased sports betting.

As mentioned earlier, gambling is strongly associated with comorbid disorders and is also considered a potentially addictive behavior that may result in related harms [26,27,38]. Problem gambling is expensive for not only gamblers and their personal networks but also for society in terms of health care and legal costs, lost productivity owing to unemployment, and poorer quality of life [39].

### Limitations

This study has some limitations. First, the data are self-reported, with the inevitable risk of recall bias. Respondents who gamble might be more likely to answer questions about it. Findings based on self-reporting would resist generalization were it not for the representative sociodemographic and geographic distribution. The advantage of our study setting is thus our use of a weighted sample: we continued to include respondents until we had a sample that was both geographically and sociodemographically representative. Another study limitation is that Sweden differs to most other countries in regard to its COVID‑19 regulations [40]. There were no lockdowns and no shopping bans because policies to prevent transmission were centered on recommendations rather than regulations, making the results difficult to transfer to other settings [40].

### Conclusions

Compared to our previous studies from earlier during the pandemic, we found that a higher proportion of the general population had increased their gambling activity during the COVID‑19 pandemic. The group that reported increased overall gambling was small but characterized by gambling problems and psychological distress. The group that reported an increase in sports betting and casino gambling were predominantly male. The group that reported an increase in casino gambling were independently related to psychological distress.

It is concerning that those who reported increased gambling activity during the pandemic are a growing group. Moreover, they are a vulnerable group that needs to be addressed by caregivers, the gambling industry, and policy makers.

We have yet to see the full effects of the COVID‑19 pandemic on gambling. More studies are needed to chart gambling patterns in possible subsequent waves of the pandemic and later in its aftermath.

## Abbreviations

OR: odds ratio
PGSI: Problem Gambling Severity Index
SEK: Swedish kronor
